# Evidence of Significant Central Fatigue in Patients with Cancer-Related Fatigue during Repetitive Elbow Flexions till Perceived Exhaustion

**DOI:** 10.1371/journal.pone.0115370

**Published:** 2014-12-22

**Authors:** Bin Cai, Didier Allexandre, Venkateswaran Rajagopalan, Zhiguo Jiang, Vlodek Siemionow, Vinoth K. Ranganathan, Mellar P. Davis, Declan Walsh, Kerong Dai, Guang H. Yue

**Affiliations:** 1 Department of Rehabilitation Medicine, Ninth People's Hospital, Shanghai Jiao Tong University School of Medicine, 639 Zhizaoju Road, Shanghai 200011, China; 2 Institute of Rehabilitation Engineering, Shanghai Jiao Tong University, 1954 Huashan Road, Shanghai 200030, China; 3 Human Performance & Engineering Laboratory, Kessler Foundation Research Center, 1199 Pleasant Valley Way, West Orange, New Jersey 07052, United States of America; 4 Department of Biomedical Engineering, Cleveland Clinic, Cleveland, Ohio 44195, United States of America; 5 Department of Physical Medicine & Rehabilitation, Cleveland Clinic, Cleveland, Ohio 44195, United States of America; 6 The Harry R. Horvitz Center for Palliative Medicine, Taussig Cancer Institute, Cleveland Clinic, Cleveland, Ohio 44195, United States of America; 7 Department of Orthopaedics, Ninth People's Hospital, Shanghai Jiao Tong University School of Medicine, 639 Zhizaoju Road, Shanghai 200011, China; 8 Engineering Research Center of Digital Medicine and Clinical Translation, Ministry of Education, PRC, 1954 Huashan Road, Shanghai 200030, China; Charité University Medicine Berlin, Germany

## Abstract

**Objective:**

To investigate whether fatigue induced by an intermittent motor task in patients with cancer-related fatigue (CRF) is more central or peripheral.

**Methods:**

Ten patients with CRF who were off chemo and radiation therapies and 14 age-matched healthy controls were enrolled. Participants completed a Brief Fatigue Inventory (BFI) and performed a fatigue task consisting of intermittent elbow-flexion contractions at submaximal (40% maximal voluntary contraction) intensity till self-perceived exhaustion. Twitch force was elicited by an electrical stimulation applied to the biceps brachii muscle. The relative degree of peripheral (muscle) vs. central contribution to fatigue induced by the intermittent motor task (IMT) was assessed using twitch force ratio (TF_ratio_) defined as post IMT twitch force to pre IMT twitch force. The total number of trials (intermittent contractions) and total duration of all trials performed by each subject were also quantified.

**Results:**

BFI scores were higher (p<0.001) in CRF than controls, indicating greater feeling of fatigue in CRF patients than controls. A significantly smaller number of trials and shorter total duration of the trials (p<0.05) were observed in CRF than control participants. The TF_ratio_ (0.81±0.05) in CRF was higher (p<0.05) compared with that of controls (0.62±0.05), suggesting CRF patients experienced a significantly lower degree of muscle (peripheral) fatigue at the time of perceived exhaustion.

**Conclusion:**

Consistent with prior findings for fatigue under submaximal sustained contraction, our results indicate that motor fatigue in CRF is more of central than peripheral origin during IMT. Significant central fatigue in CRF patients limits their ability to prolong motor performance.

## Introduction

Cancer-related fatigue (CRF), a persistent subjective sense of tiredness related to cancer and/or its treatment, interferes with daily activities and worsens quality of life of patients [1]. CRF is one of the most common symptoms experienced by cancer patients, with most studies reporting prevalence rates above 60% and some studies reporting rates of up to 90% [2]. CRF is characterized by a feeling of fatigue or tiredness greater than what would be expected for a given activity or under a given condition, which leads to distress, reduced ability to participate in activities of daily living (ADL), diminished quality of life, and physical and psychological well-being [3]–[9]. It is important, therefore, to understand the underlying pathophysiological mechanisms of CRF in order to develop strategies for more accurate and objective diagnosis and treatment of the condition.

So far, the underlying pathophysiology of CRF has not been adequately elucidated [10]. Although a number of hypotheses regarding mechanisms of CRF have been proposed, these hypotheses have been largely based on evidence where fatigue is an intrinsic characteristic, such as in chronic fatigue syndrome and daily activity/exercise [11]. Very few studies have evaluated peripheral and central contributions to fatigue induced by a specific task or condition in CRF such as muscle fatigue caused by a long bout of exercise. It has been reported that altered neuromuscular conditions including muscle fatigue or impaired endurance associated with cancer could contribute to CRF [12]–[16].

It is well known that muscle fatigue, any exercise-induced reduction in the ability of muscle to produce force or power [17]–[20], has a central and/or peripheral origin [21]. A direct measure of peripheral (muscle) fatigue is the change of force generating ability of muscle before and after the motor fatigue task by evaluating electrical stimulation-elicited twitch force at rest.

Since the twitch force generation is independent of the central motor drive, comparison of its change with the changes in maximum voluntary contraction also provides an estimate of the central fatigue. To the best of our knowledge, very few studies have ever attempted to evaluate mechanisms of muscle fatigue in patients with CRF. Our previous studies in CRF showed that muscle fatigue resulting from continuously sustaining a submaximal force/load at elbow joint till self-perceived exhaustion was contributed more by central than peripheral mechanisms [14], [15]. However, in healthy individuals, the development of central or peripheral fatigue is task-dependent [21]. Furthermore, our functional magnetic resonance imaging study has shown unique brain activation patterns between fatigue tasks of sustained and intermittent submaximal muscle contractions, i.e., different cortical activation patterns, and perhaps cell populations are involved in controlling sustained and intermittent voluntary motor activities. [22] These findings suggest that intermittent vs. sustained task induced fatigue may have a different underlying mechanism in CRF. Thus, the aim of the current study is to assess whether greater central-than-peripheral fatigue in CRF still holds for intermittent motor task, which more closely resembles activities of daily living. We hypothesized that similar to the sustained motor task, the intermittent motor task (IMT) would induce greater central than peripheral fatigue in CRF patients compared to healthy controls.

## Methods

### Subjects

Ten CRF patients with solid cancer (lung, kidney, liver, thyroid, breast, and gastrointestinal cancer, more details about the cancer information can be found in our previous study [14]) and fourteen healthy controls were enrolled. Even though we used the same population as our prior studies on sustained contraction [14], the number of patients in the current study was slightly different since datasets for some subjects were excluded from the analysis due to the dynamic (intermittent) contractions causing excessive artifact contaminations to the force, EEG and EMG data (EEG & EMG results are not presented in this paper).The median age of the cancer patients was 58.8 years (range: 48–82 years, 3 males) and that of the controls was 51.5 years (range: 26–72 years, 5 males). There was no significant difference in age (p = 0.18) or body mass index (BMI) (p = 0.56) between the CRF patients and controls. The inclusion criteria for this study were that the patients should not have received any chemotherapy or radiation therapy for at least four weeks prior to the study and had to be at least four weeks postoperative. Patients were screened using the brief fatigue inventory (BFI) [23], a nine-item self-assessment questionnaire. Patients and controls were also screened by a single question, “Are you depressed?”. Anyone who answered “yes” to the question was excluded. Eligible patients had a hemoglobin concentration of more than 10 g/dL, and no evidence of polyneuropathy, amyotrophy, or a myasthenic syndrome by history or physical examination. Patients with more than 10% of pre-illness body weight loss or with significant pulmonary compromise defined by oxygen dependence were also excluded. Healthy controls were recruited through local advertisement. All study procedures were approved by the Cleveland Clinic Institutional Review Board and all the subjects provided written informed consent.

### Experimental Protocol

The experimental procedures were as follows: (1) Participants first completed the BFI. (2) Maximum voluntary contraction (MVC) force of isometric elbow flexion was measured. Before the measurement of MVC, participants performed two trials of submaximal elbow contraction (roughly 30–40% perceived maximal effort) for few seconds as warm-up activities and to familiarize themselves with the isometric contraction task. (3) Maximum electric stimulation-evoked twitch force (TF) at rest before the task (TF_pre-fatigue_) was recorded. (4) Participants performed the IMT consisting of repetitive elbow flexion contractions at 40% MVC until subjective exhaustion. (5) Immediately after the IMT, evoked TF (TF_post-fatigue_) and MVC force were measured again.

### MVC Force

Isometric elbow flexion MVC force of the dominant arm was measured by a force transducer (JR3 Universal Force-Moment Sensor System, Woodland, CA), with subjects seated, forearm in a neutral position (between supination and pronation), and an elbow joint angle of ∼100^°^. Participants were encouraged to exert maximal strength. The MVC force was displayed on an oscilloscope and recorded onto a personal computer using a data acquisition system (1401 Plus, Cambridge Electronic Design, Ltd., Cambridge, UK). Two MVC measurements were made before the fatigue exercise and the highest value was used for analysis. If the percentage difference in force between the two measurements was more than 5%, a third measurement was performed to ensure that true maximum force was achieved. Only one MVC trial was performed immediately after the fatigue task. More details about the MVC measurement can be found in our previous study [14].

### Motor Fatigue Task

All participants performed an IMT to fatigue the elbow flexor muscles. The IMT consisted of intermittent contractions to a pre-determined target level (40% MVC). Each contraction lasted 5 seconds with a 2-second rest between trials. Subjects followed visual cues projected onto the oscilloscope in front of them to time initiation and termination of each contraction. They kept performing the repeated contractions till self-perceived exhaustion. Those who did not feel extremely fatigued following 30 min of the IMT (>250 contractions) were asked to perform a sustained contraction till exhaustion (this was done by 12 controls and 1 CRF patient).

### Twitch Force at Rest

Force generating ability of muscle by evaluating electrical stimulation-elicited twitch force (TF) is an accurate, objective and widely used method for direct assessment of muscle (peripheral) fatigue [24]. TF is obtained by performing a supra-maximal stimulation of the muscle belly or motor nerve going into the muscle and measuring the resulting generated force, hence assessing the alteration in the intrinsic motoneuron excitation to force output transfer properties of the muscle associated with peripheral fatigue [17], [19], [24]. Maximal TF_pre-fatigue_ was assessed by attaching stimulation electrodes to the skin over the biceps brachii muscle, one of the major elbow flexors. One electrode (anode) was attached to the skin above the tendon proximal to the elbow and another (cathode) was placed over the muscle belly or motor point. The dimension of each rubber electrode was 5×5 cm^2^. TF was evoked by applying a single supra-maximal-intensity electrical pulse (1-ms duration) generated by a digital stimulator (Grass S8800, Astro Med Inc., West Warwick, RI). The resulting TF was measured with the same force transducer used to obtain MVC. Immediately after IMT, the TF was assessed again under fatigue condition (TF_post-fatigue_). The TF was measured from the baseline to its peak and normalized to the pre-fatigue MVC force. This normalized TF provides a more sensitive assessment of muscle fatigue than its absolute value [15].

### Data Processing

Data was first visually reviewed to eliminate bad trials. We determined duration of each trial by identifying beginning and end of the contraction - the time when the force crossed the half target force amplitude (set at 40% MVC) during the ascending phase was defined as beginning of the trial and that crossed the same force level during descending phase was defined as end of the contraction. A trial was rejected when its duration was shorter than 1.3 seconds and/or when the force failed to reach 70% of the target force amplitude. Good trials were defined as those with trial duration between 3 and 6 seconds and with no twitch force elicited during the contraction (which was performed once every min or 1 trial with interpolated twitch in about every 8 trials). The first and last two trials were also not included to remove transient phase in and out effects. We then computed trial-averaged outcomes at the beginning, middle and end of the task corresponding stages of mild, moderate and severe fatigue. This was accomplished by first dividing the total number of trials equally into three blocks, corresponding to assumed mild, moderate and severe fatigue stages. Subsequently, outcomes were measured for the 20 contiguous good (as defined above) contraction trials within each of the three blocks as follows: (i) the first 20 trials in the first block to assess fatigue at the early stage of the task, (ii) the middle 20 trials in the second block to assess fatigue at the half point of the task, and (iii) the last 20 trials in the third block to assess fatigue at the point of perceived exhaustion. All force measures were normalized to each subject's pre-IMT MVC force.

### Outcome Measures

The following outcomes were measured for each subject: (i) MVC force as the measure of strength; (ii) total number of trials and total duration of all the trials (including the last SC) as measures of endurance (since some subjects in the study group performed a SC at the end which was significantly longer than an intermittent trial, the total number of trials was computed so that it equals the number of intermittent trials plus the number of “equivalent” trials resulting from dividing the duration of the SC trial by the mean duration of the intermittent trials.); (iii) mean trial duration based on all the trials (except the SC at the end performed by some subjects) as a measure of trial time precision; and (iv) pre- and post-task normalized twitch forces (TF_pre-fatigue_ and TF_post-fatigue_) to obtain post-to-pre-fatigue TF ratio (TF_ratio_) as measures of muscle or peripheral fatigue. We also assessed changes in motor performance and accuracy by computing trial-averaged values of the following outcomes for the first, middle and last 20 trials: (v) average normalized (to MVC) force amplitude during the plateau phase of the contraction (between time 0.5 s and 2 s from contraction onset as defined above corresponding to early static contraction phase after the force reached the target); (vi) average normalized (to MVC) force area within the force curve; and (vii) average force slope during ascending and descending phases of the force curve as a measure of contraction and relaxation speed. The slope was computed by first applying a zero-phase 4^th^ order Butterworth 10 Hz low pass filter to the force and then, measuring the maximum of 2^nd^ order polynomial fit of the bell-shaped maximum of the absolute force derivative during the ascending and descending phases of the force curve.

### Statistical Analysis

Data normality was first checked using the Kolmogorov-Smirnov test and by looking at the histogram plots and skewness and kurtosis values to decide whether to perform a parametric or non-parametric analysis. For twitch force, non normality was mainly caused by a single outlier, which was removed before performing parametric analyses.

MVC force, number of trials and total duration of trials were found to have a non-normal distribution and thus analyzed using Mann-Whitney U-test for between and Wilcoxon for within group comparisons. One-sample t-test (compared to 1 i.e. no pre to post changes) for each group and independent sample t-test were performed on MVC force_ratio_ and TF_ratio_ to study within and between group fatigue effect.

A two-way (group x time) repeated measures ANOVA using general linear model (GLM) was applied to study the effect of fatigue on normalized twitch force, normalized (target) force, mean trial duration, normalized force area, and ascending and descending force slopes. When the sphericity assumption using Mauchly's test failed, the multivariate rather than the univariate results were reported. Whenever an overall significant effect was found, corresponding post-hoc analyses were performed to examine within and between group changes over time (middle and end vs. the beginning of the IMT or post vs. pre IMT). Between-group baseline (beginning of the task or before IMT) comparisons were also performed using simple t-test. Due to the exploratory nature of the study, no sample size calculation and correction for multiple comparisons were made.

Unless otherwise stated, results were reported using mean and standard error of the mean (SEM) for parametric results and, median and 25^th^ - 75^th^ percentiles for non-parametric results. Statistical significance level was set at p≤0.05. All statistical analyses were performed using IBM SPSS Statistics 21.

## Results

### BFI

Mean BFI score for nine questions was significantly higher in CRF patients (mean and SD of 4.69±2.05) than controls (0.91±1.02) (p<0.001) indicating that subjective feeling of fatigue was substantially greater for CRF than control participants.

### MVC Force

**Figure 1:**
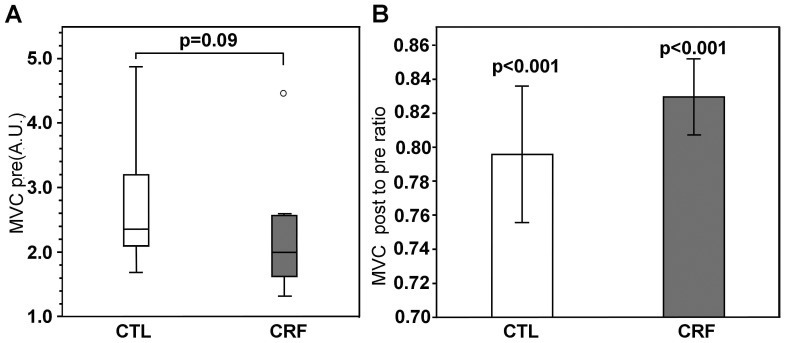
Baseline MVC force (A) and post-to-pre-IMT MVC force ratio (B) in CRF patients and controls. CRF  =  cancer-related fatigue, CTL  =  Controls. The circle in A represents mild outliers (extending beyond 1.5 IQR from the IQR box edges).

Pre-IMT MVC force was somewhat lower in CRF patients than controls (median [25^th^; 75^th^ percentiles] of 2.0 [1.7; 2.51] compared to 2.35 [2.12; 3.11], U = 41, p = 0.09). Within group comparison using Wilcoxon test showed a significant pre to post decrease in MVC force for both CRF (−0.29 [−0.37; −0.23], T = 5, p = 0.03) and controls (−0.51 [−0.79; −0.25], T = 0, p = 0.005). However, the MVC force decreases after vs. before the IMT were not significantly different between the two groups (U = 52, p = 0.29) (Fig. 1-A).

Similarly, the post-to-pre-IMT MVC force ratio was significantly less than 1 (i.e. no change) for both controls (mean ± SEM of 0.80±0.04, t(13) = −5.09, p<0.001) and CRF (0.83±0.02, t(9) = −7.62, p<0.001) participants, suggesting a significant decrease in the maximal voluntary force output due to fatigue (Fig. 1-B). Between-group comparison of the MVC force ratios was non-significant (−0.03±0.05, t(19.6) = −0.74, p = 0.47).

### Intermittent Motor Task (IMT) Related Measures

#### Number and duration of trials

**Figure 2:**
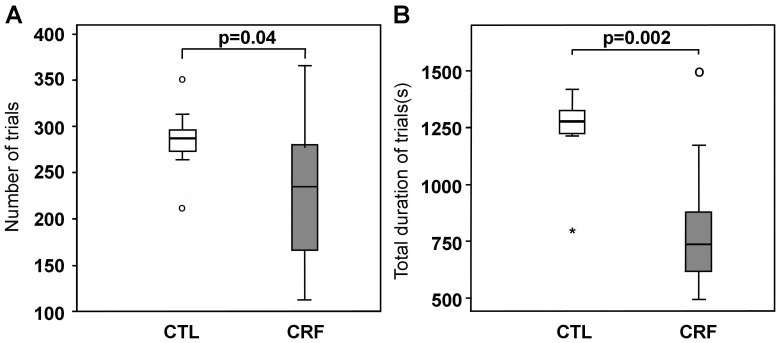
Number of trials (A) and total duration of trials (B) in CRF patients and controls. **CRF** =  cancer-related fatigue, CTL  =  Controls. Circles and stars represent mild and extreme outliers respectively (extending beyond 1.5 and 3 IQR from the IQR box edges).

**Figure 3:**
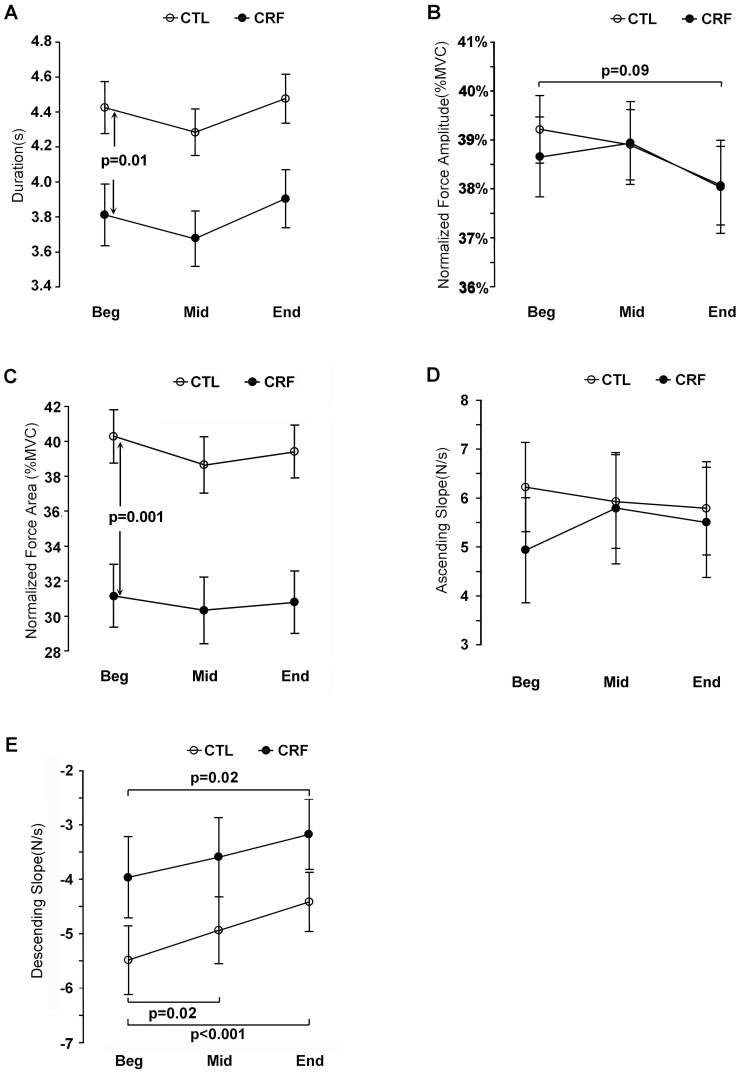
Mean duration (A), normalized force amplitude (B), normalized force area (C), ascending slope (D) and descending slope (E) over 20 trials at the beginning (Beg), middle (Mid) and end (End) blocks of the IMT representing mild, moderate and severe fatigue stages, respectively in controls (open circles) and CRF patients (filled circles). CRF  =  cancer-related fatigue, CTL  =  control.

The number of trials (intermittent elbow flexion contractions) was significantly smaller in CRF than controls (235.5 [168; 278] vs. 268 [260; 276], U = 35, p = 0.04) (Fig. 2-A). In addition, mean contraction (trial) duration at the beginning of the IMT was significantly shorter for CRF compared to controls (3.81±0.18 sec vs. 4.42 ±0.15 sec, t(22) = 7.04, p = 0.01). Given that the GLM found no time (F(2,44) = 2.3, p = 0.113) and no group by time effect (F(2,0) = 0.03, p = 0.975), the shorter trial duration in CRF than controls persisted throughout the IMT (Fig. 3-A). The combined lower number and shorter duration trials resulted in a significantly shorter total cumulated contraction duration of all the trials for CRF (736 [621; 871] sec vs. controls 1277 [1226; 1324] sec, U = 17, p = 0.002) (Fig. 2-B).

#### Normalized force amplitude

GLM analysis did not find any significant time (F(2,44) = 2.05, p = 0.14) or group by time effect (F(2,0) = 0.22, p = 0.81) for normalized force amplitude (CRF 38.6%±0.8% vs. controls 39.2%±0.7%). Post-hoc analysis revealed a marginal decrease in force at the end compared to the beginning of the IMT for controls (t(22) = 1.8,p = 0.09) (Fig. 3-B).

#### Normalized force area

For normalized force area, GLM analysis indicated no time (F(2,21) = 1.6, p = 0.22) or group by time effect (F(2,21) = 0.17, p = 0.85) (Fig. 3-C). However the CRF group showed a significantly lower force area at the beginning of the task compared to controls (CRF 31.2±1.8 vs. CTL 40.3±1.5, t(22) = 14.91, p = 0.001).

#### Ascending slope

There was no significant time (F(2,21) = 0.9, p = 0.42) or group by time effect (F(2,21) = 0.76, p = 0.48) from the GLM analysis and no between group difference at the beginning of the task for the ascending force slope (Fig. 3-D).

#### Descending slope

For the descending force slope, GLM analysis revealed a time (F(2,21) = 16.7, p<0.001) but no group by time effect ((F2,0) = 0.33, p = 0.721) (Fig. 3-E). Post-hoc analyses for within group comparisons find a significant decrease in absolute slope value over time for CRF (from 3.96±0.75 at the beginning down to 3.59±0.73 in the middle of the task, t(22) = 1.21, p = 0.24, and 3.17±0.65 at the end of the task, t(22) = 2.64, p = 0.01) and controls (from 5.48±0.63 to 4.93±0.61, t(22) = 2.11, p = 0.05 in the middle and 4.41±0.55, t(22) = 4.21, p<0.001 at the end of the task).

#### Normalized Twitch Force

**Figure 4:**
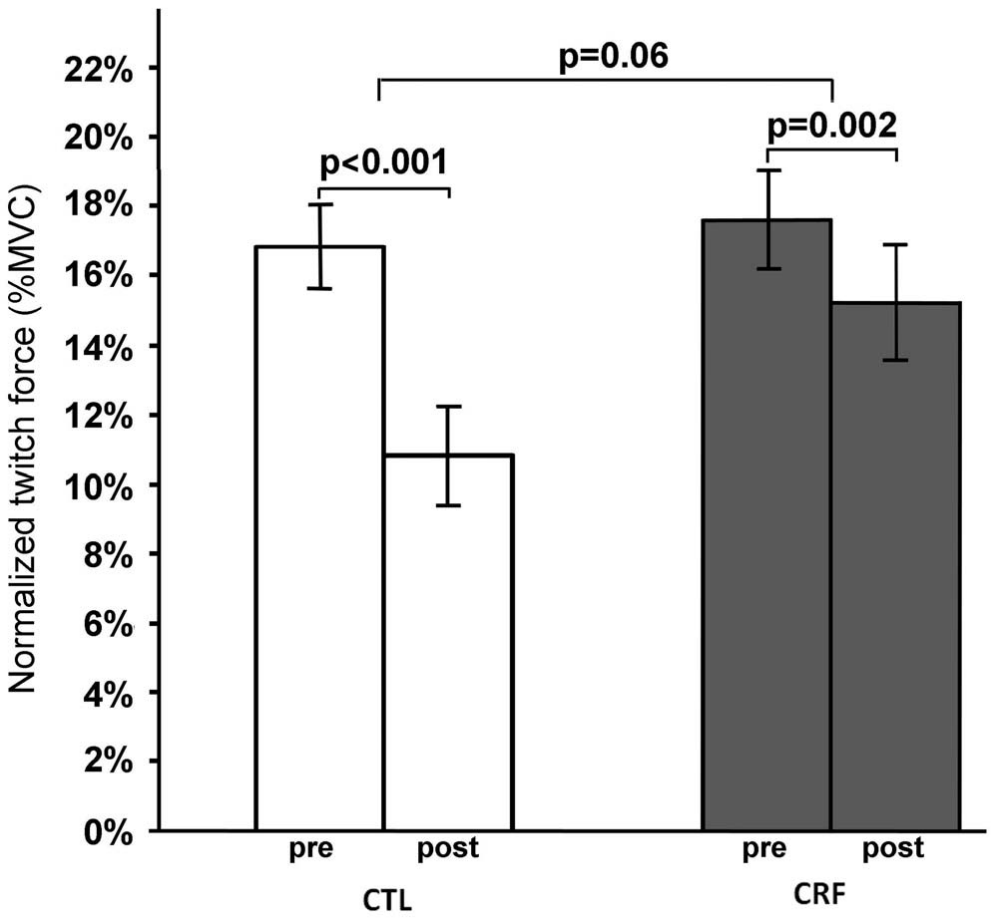
Normalized (%MVC) pre- and post-IMT twitch forces in CRF patients and controls. CRF  =  cancer-related fatigue, CTL  =  control.

Repeated ANOVA analysis showed a time (F(1,21) = 57.96, p<0.001) effect for the normalized twitch force. Post-hoc tests found that the twitch force was significantly reduced both for controls (from 16.8%±1.2% down to 10.8%±1.3%, t(21) = 7.69, p<0.001) and CRF (17.6%±1.5% down to 14.1%±1.6%, t(21) = 3.59, p = 0.002) patients (Fig. 4). The marginal significant group by time effect ((F1,21) = 4.03, p = 0.06) indicates that this reduction was greater in the control group.

#### Twitch Force Ratio

**Figure 5:**
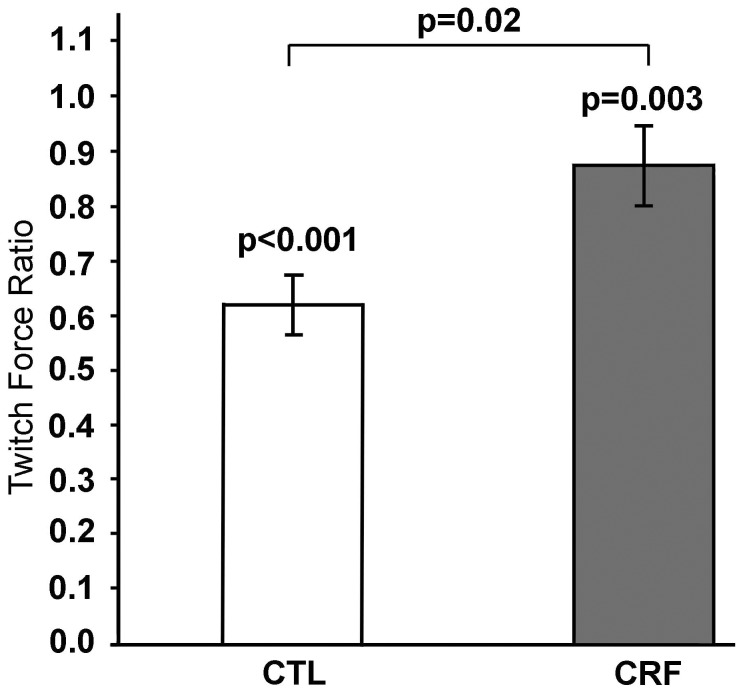
Post-to-pre-IMT twitch force ratio in CRF patients and controls. CRF  =  cancer-related fatigue, CTL  =  control.

TF_ratio_ was found to be significantly less than 1 (i.e. no change) for both controls (TF_ratio_ = 0.62±0.05, t(13) = −7.03, p<0.001) and CRF patients (0.81±0.05, t(8) = −4.13, p = 0.003). Between group comparison analysis also indicates that TF_ratio_ in CRF group was significantly higher than in controls (t(21) = −2.52,p = 0.02) (Fig. 5), indicating force generating capability of the muscle immediately after the IMT or fatigue was greater in CRF than controls, which suggests a lower degree of muscle (peripheral) fatigue at the time of perceived exhaustion.

## Discussion

This study hypothesized that by performing a long-duration intermittent motor task (IMT), CRF patients would endure greater central fatigue when compared to healthy controls. Our primary findings supporting this hypothesis include (i) CRF patients had greater perceived fatigue (significantly higher BFI score when compared to controls) and (ii) the TF_ratio_ was significantly higher in CRF patients compared to that of healthy controls.

We chose intermittent submaximal task in present study. Submaximal motor task is considered to be a more realistic and thus a more appropriate model to study central fatigue than the less widely used maximal force protocol [21]. Submaximal force exertions are a more representative of activities of daily living (ADL) and it has been shown that CRF patients experience more fatigue due to ADL [5]. Also during a submaximal force protocol, adequate blood and oxygen are refilled to the muscle during and between contractions to prevent local accumulation of metabolites that cause peripheral fatigue. In addition, submaximal exercise is associated with aerobic metabolism and predominantly recruits fatigue-resistant muscles/motor units to avoid/delay muscle fatigue. For the above reasons submaximal motor tasks seem to be more appropriate for studying motor-related fatigue in CRF. Secondly, Two types of submaximal motor activities are widely adopted to study motor fatigue, sustained and intermittent exercises. However, intermittent submaximal motor activities mimic more closely ADL compared with a long-duration sustained muscle contraction. To our best knowledge, no study has used intermittent submaximal motor task to investigate central and peripheral contributions to CRF.

The performance of IMT revealed that CRF patients were only able to exert a smaller number of trials as well as shorter total duration of trials than healthy controls. This finding indicates that CRF patients felt exhausted (fatigued) significantly faster than controls. This is despite the fact that CRF patient exerted themselves less at each trial as evidenced by the lower normalized force area (a measure of relative average force output per trial) and shorter mean trial duration and thus longer resting time between trials (given the contraction-rest cycle set 7 sec for both groups). Furthermore, it is also important to consider that CRF patients were weaker (lower MVC force) and thus performed the task at a lower absolute target force than controls. These findings taken all together mean that the patients performed a smaller number of motor activities for a shorter total duration against an even lighter load than controls. This fact does suggest that CRF patients face greater challenges in performing real-life tasks such as carrying a case of bottled water or grocery bags from the driveway into the house, emphasizing the importance of alleviating this debilitating condition.A greater post-to-pre IMT TF_ratio_ in CRF compared to controls provides evidence suggesting that the earlier feeling of severe fatigue which resulted in the performance of a smaller number of motor trials and shorter total exertion time was largely a consequence of worsened central fatigue in patients. More specifically, the higher TF_ratio_ indicates that the force generating capability of the muscle in CRF was not as much deteriorated as in controls, which suggests that patients endured a lower degree of muscle fatigue at the time of perceived exhaustion. Thus, if muscle fatigue was not the primary contributor to earlier failure of the IMT (a smaller number of trials and shorter total trial duration) in CRF patients, we can confidently argue that the poorer motor performance was caused by a greater level of central fatigue.

Although the spinal and supraspinal factors in muscle fatigue has been well studied [24], the mechanism for the pathophysiology of CRF is still not clear [10]. Central fatigue, which develops in the central nervous system (CNS), arises from the progressive failure of transmitting motor neuron impulses [24]. Central fatigue has been defined as difficulty in the initiation or maintenance of voluntary activities [25]. Large experimental evidence indicates that voluntary motor drive plays an important role in the origin of fatigue [26]–[30]. Central fatigue can originate at spinal and supra-spinal sites [24]. Spinal regulation mainly involves the control of alpha and gamma motor neuron activities by a number of mechanisms. Supra-spinal regulation is based not only on the activity of primary motor cortex, but also on the function of the structures involved in planning and control of movement and on interaction among various levels of motor control network. In CRF patients, these regulations may be impaired due to dysregulation of several physiological and biochemical systems [11], [31]. In particular, it is known that cancer and/or cancer treatment causes an increase in brain serotonin (5-HT) levels and/or up-regulation of a population of 5-HT receptors, leading to reduced somatomotor drive, modified hypothalamic–pituitary–adrenal (HPA) axis function, and a sensation of reduced capacity to perform physical work [32]. Studies have reported reduced cortisol response in cancer survivors with persistent fatigue [33], [34]. These findings may explain in part why the CRF patients experience more central than peripheral fatigue in motor performance. Future studies should elucidate underlying causes of central fatigue in CRF, including the role of 5-HT and cortisol in regulating descending drive for muscle activation.

Better knowledge of the mechanisms of CRF or any of its components would greatly assist in developing targeted interventions [35], [36]. Understanding the origins of fatigue during ADL in cancer survivors is particularly important for accurate diagnosis and effective treatment of CRF. Because motor-related CRF results largely in a reduction of central motor drive [14], fatigue management strategies should focus on improving central as well as peripheral fatigue. Moreover, the administration of central stimulant drugs to patients with CRF may be crucial during treatment. These data are preliminary and the relatively small sample size of the study may limit generalization of the results to all cancer survivors.

## Conclusions

Consistent with prior findings for fatigue under submaximal sustained contraction, our findings support that CRF, especially associated with motor performance is more of central rather than peripheral origin. Thus, a large discrepancy exists between perceived physical exhaustion and the true physiological condition of the muscle at the time of task failure in CRF patients. Central fatigue in CRF patients is a significant contributor to the limitation of their ability to prolong motor performance.

## Supporting Information

S1 Dataset **Full manuscript dataset.** This excel file provides the full dataset of the manuscript. The file has two worksheets; “AllData” containing the dataset for all outcome variables and “Variables” providing a variable name dictionary. Variable names highlighted in red are those of outcomes used and reported in the manuscript.
(XLSX)
